# Addressing challenges in radiomics research: systematic review and repository of open-access cancer imaging datasets

**DOI:** 10.1186/s13244-023-01556-w

**Published:** 2023-12-12

**Authors:** Piotr Woznicki, Fabian Christopher Laqua, Adam Al-Haj, Thorsten Bley, Bettina Baeßler

**Affiliations:** 1 Department of Diagnostic and Interventional Radiology, University Hospital Würzburg, Würzburg, Germany; 2 Faculty of Medicine, Medical University of Warsaw, Warsaw, Poland

**Keywords:** Radiomics, Radiology, Cancer imaging, Machine learning, Reproducibility of results

## Abstract

**Objectives:**

Open-access cancer imaging datasets have become integral for evaluating novel AI approaches in radiology. However, their use in quantitative analysis with radiomics features presents unique challenges, such as incomplete documentation, low visibility, non-uniform data formats, data inhomogeneity, and complex preprocessing. These issues may cause problems with reproducibility and standardization in radiomics studies.

**Methods:**

We systematically reviewed imaging datasets with public copyright licenses, published up to March 2023 across four large online cancer imaging archives. We included only datasets with tomographic images (CT, MRI, or PET), segmentations, and clinical annotations, specifically identifying those suitable for radiomics research. Reproducible preprocessing and feature extraction were performed for each dataset to enable their easy reuse.

**Results:**

We discovered 29 datasets with corresponding segmentations and labels in the form of health outcomes, tumor pathology, staging, imaging-based scores, genetic markers, or repeated imaging. We compiled a repository encompassing 10,354 patients and 49,515 scans. Of the 29 datasets, 15 were licensed under Creative Commons licenses, allowing both non-commercial and commercial usage and redistribution, while others featured custom or restricted licenses. Studies spanned from the early 1990s to 2021, with the majority concluding after 2013. Seven different formats were used for the imaging data. Preprocessing and feature extraction were successfully performed for each dataset.

**Conclusion:**

RadiomicsHub is a comprehensive public repository with radiomics features derived from a systematic review of public cancer imaging datasets. By converting all datasets to a standardized format and ensuring reproducible and traceable processing, RadiomicsHub addresses key reproducibility and standardization challenges in radiomics.

**Critical relevance statement:**

This study critically addresses the challenges associated with locating, preprocessing, and extracting quantitative features from open-access datasets, to facilitate more robust and reliable evaluations of radiomics models.

**Key points:**

- Through a systematic review, we identified 29 cancer imaging datasets suitable for radiomics research.

- A public repository with collection overview and radiomics features, encompassing 10,354 patients and 49,515 scans, was compiled.

- Most datasets can be shared, used, and built upon freely under a Creative Commons license.

- All 29 identified datasets have been converted into a common format to enable reproducible radiomics feature extraction.

**Graphical Abstract:**

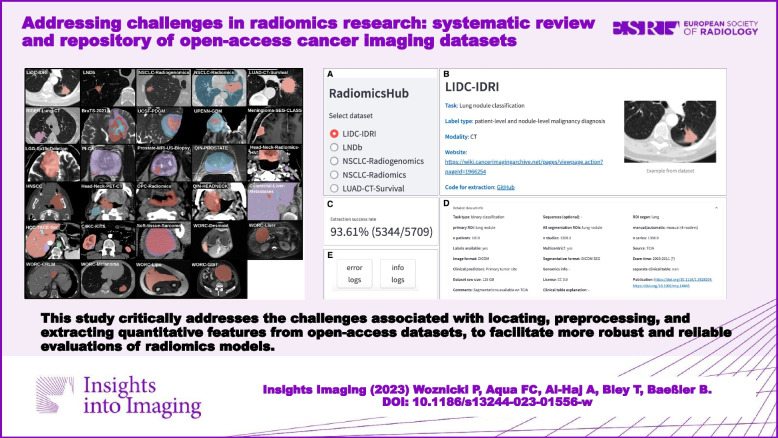

**Supplementary Information:**

The online version contains supplementary material available at 10.1186/s13244-023-01556-w.

## Introduction

The advent of quantitative image analysis techniques has revolutionized the field of radiology, enabling researchers and clinicians to analyze and interpret medical imaging data more efficiently and accurately [1]. Radiomics, an emerging field at the intersection of radiology and oncology, leverages the power of advanced computational techniques to extract a wealth of quantitative information from different imaging modalities [2]. This process involves extracting numerous high-dimensional features that capture various aspects of the tumor and its surrounding microenvironment, including shape, size, texture, intensity, spatial relationships, and heterogeneity within the tumor [3].

By converting medical images into mineable, high-dimensional data, radiomics can uncover potential biomarkers that can aid in various aspects of cancer diagnosis, prognosis, monitoring treatment response, and personalizing therapy plans according to each patient’s individual needs. In the domain of oncologic imaging, quantitative analysis using labeled cross-sectional imaging data to guide the model has arguably seen the biggest success, with applications ranging from organ segmentation [4] and lesion detection [5] to cancer characterization and risk stratification [6]. Furthermore, radiomics can potentially improve the efficiency and cost-effectiveness of cancer care by reducing the need for invasive biopsies and enabling earlier detection of malignancies [7]. By providing non-invasive, quantitative, and reproducible information, radiomics can complement traditional imaging techniques and contribute to a more comprehensive understanding of a patient’s cancer and its underlying biology [8].

Unlike deep learning approaches, which are capable of learning features and patterns directly from raw image data, radiomics typically requires predefined regions of interest (ROIs) to be segmented within the dataset. As a result, radiomics approaches require additional preparatory steps to delineate relevant areas for later feature extraction and analysis. However, because labeling is expensive and time-consuming, datasets containing both accurate inputs and labels are often difficult to find and often reused as benchmarks across many different studies [1]. Open-access datasets have emerged as an invaluable resource for validating new radiomics approaches, providing researchers with diverse and annotated data [9].

Despite the growing number of publicly available datasets, numerous challenges hinder their effective utilization in radiomics research. These issues include incomplete documentation, low visibility, inconsistency in image and segmentation formats, data inhomogeneity across disparate datasets, and complex data preprocessing. Inadequate documentation and mislabeling in datasets can lead to misinterpretation and unintentional bias, whereas low visibility stems from datasets being hosted across various platforms. The absence of centralized data repositories with standardized formats impedes system interoperability and limits opportunities for collaboration and shared progress in the field. Furthermore, differences in acquisition protocols, scanners, and settings across studies can introduce bias and diminish the robustness of radiomics models. Depending on the clinical application, datasets might require custom, time-consuming preprocessing to handle multiple modalities (e.g., CT and PET), sequences, ROIs, or readers and to verify data correctness before their use in a radiomics analysis.

The lack of reproducibility and generalizability of radiomics models is another major challenge. Insufficient transparency in reporting radiomics studies further prevents the translation of the developed radiomics signatures into clinical practice. In recent years, several notable efforts to improve reproducibility and standardization in radiomics studies have been initiated, including the Image Biomarker Standardization Initiative (IBSI) [10], which identified a reference set of reproducible radiomics features, and the CheckList for EvaluAtion of Radiomics Research (CLEAR) [11], which provided guidelines for more structured and consistent documentation for radiomics studies. While these initiatives primarily focused on improving study methodologies, access to high-quality, open-source data is the other crucial element for further progress in the field.

In this study, we systematically reviewed cross-sectional cancer imaging datasets, specifically identifying those suitable for radiomics research. We created a code repository and curated a comprehensive data repository to facilitate the evaluation of new radiomics models on benchmark datasets, addressing the time-consuming task of locating appropriate datasets with segmentations and clinical labels and preprocessing them from their raw form. We hope that the project will catalyze further advancements in this field, promoting standardization, reproducibility, and ultimately the clinical translation of radiomics research.

## Methods

### Dataset selection and acquisition

**Fig. 1:**
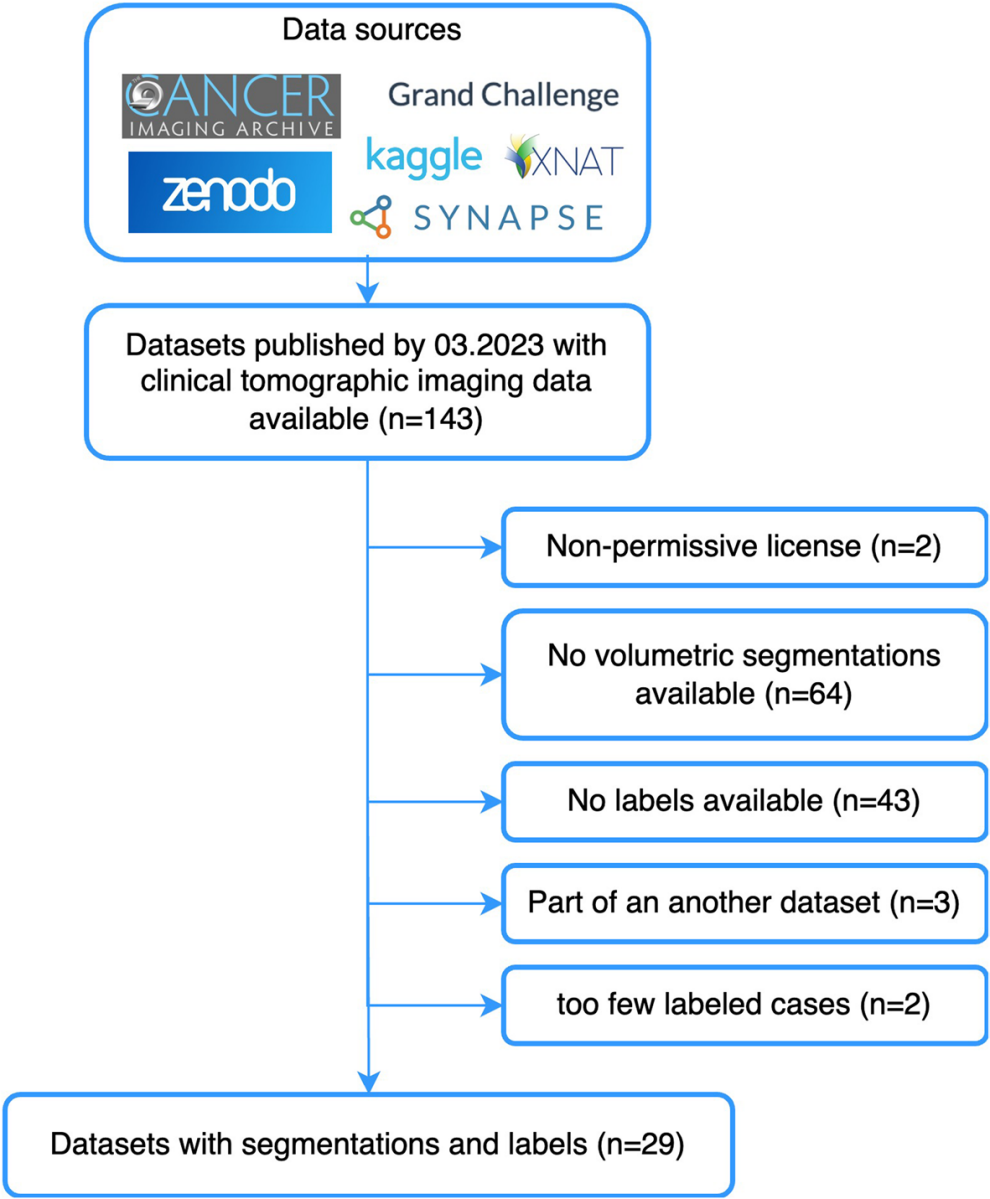
Study flowchart with the inclusion and exclusion criteria

We reviewed multiple publicly available imaging datasets spanning various oncologic entities. The datasets were acquired from established online data repositories and included The Cancer Imaging Archive (TCIA) [12], the Grand Challenge platform (https://grand-challenge.org, Radboud University Medical Center, 2023), Zenodo [13] (https://zenodo.org), Synapse (https://synapse.org, Sage Bionetworks, 2023), and BMIAXNAT [14]. Inclusion criteria encompassed (1) dataset publication on one of the abovementioned repositories by March 2023 and (2) availability of a tomographic imaging modality (CT, MRI, or PET). Exclusion criteria included the following: non-permissive license, absence of volumetric (3D) segmentations, unavailability of clinical labels, the dataset being included in another public dataset, and an insufficiently small number of labeled cases (*n* < 10). The study flowchart, displaying data sources as well as the inclusion and exclusion criteria, is presented in Fig. 1. Labels were defined as clinical outcomes, characteristics, or classifications related to the imaging data. These can be used to guide a machine learning model to learn the mapping from radiomics features to clinical information by examining labeled examples. The labels are therefore necessary for developing clinical radiomics models. We also collected detailed information about each dataset, including its clinical task, imaging modality, cohort size, data format, region of interest, annotation process, label availability, and license.

### Data preprocessing

Raw data were acquired from various sources in different formats, including DICOM (Digital Imaging and Communications in Medicine), NIfTI (Neuroimaging Informatics Technology Initiative), MetaImage, and others. Data preprocessing was performed to ensure uniformity and compatibility across all datasets for subsequent analysis. This process involved converting image and segmentation formats into NifTI, intensity normalization, and resampling to a common voxel size. Preprocessing parameters are described in detail in Supplement S1. Standard Python libraries, including SimpleITK [15], NiBabel [16], and PlatiPy [17], were used for processing volumetric medical imaging data. Multichannel images were split into separate volumetric images, and segmentations with multiple labels (e.g., for multiple ROIs, organs, or readers) were split into separate segmentations. Modalities, ROI names, and readers were explicitly encoded in the filename as well as in the tables containing relevant metadata. Each image-segmentation pair was assigned a unique ID to streamline subsequent feature extraction. Data identified as corrupt were excluded, with all associated errors carefully logged.

### Feature extraction

Radiomic features were extracted from the segmented regions of interest using standardized methods. Included radiomics feature classes were selected from the standardized set of features validated in the Image Biomarker Standardization Initiative [10] and included first-order statistics, 3D shape-based features, and texture features derived from the Gray Level Size Zone (GLSZM), Gray Level Dependence Matrix (GLDM), Gray Level Co-occurrence Matrix (GLCM), Neighbouring Gray Tone Difference Matrix (NGTDM), and Gray Level Run Length Matrix (GLRLM). Feature extraction was performed using the open-source *AutoRadiomics* [18] framework, which performs the standard extraction based on the *pyradiomics* [19] library. Extraction parameters are detailed in Supplement S2.

All the processing steps for each dataset were run as a single script using Python 3.10 and are documented in the code repository at https://github.com/pwoznicki/RadiomicsHub. The repository is distributed under the permissive MIT license.

### Dataset repository

We have built a dedicated website for the project, which conveniently presents all the extracted metadata for each dataset, along with tables of radiomics features, clinical data, and labels. It can be accessed at https://radiomics.uk. The website provides backlinks to the original data sources and references to studies that have used each dataset. Radiomics features and clinical parameters can be directly downloaded and used to develop machine-learning models for the prediction of specific clinical outcomes.

## Results

### Dataset overview

**Fig. 2:**
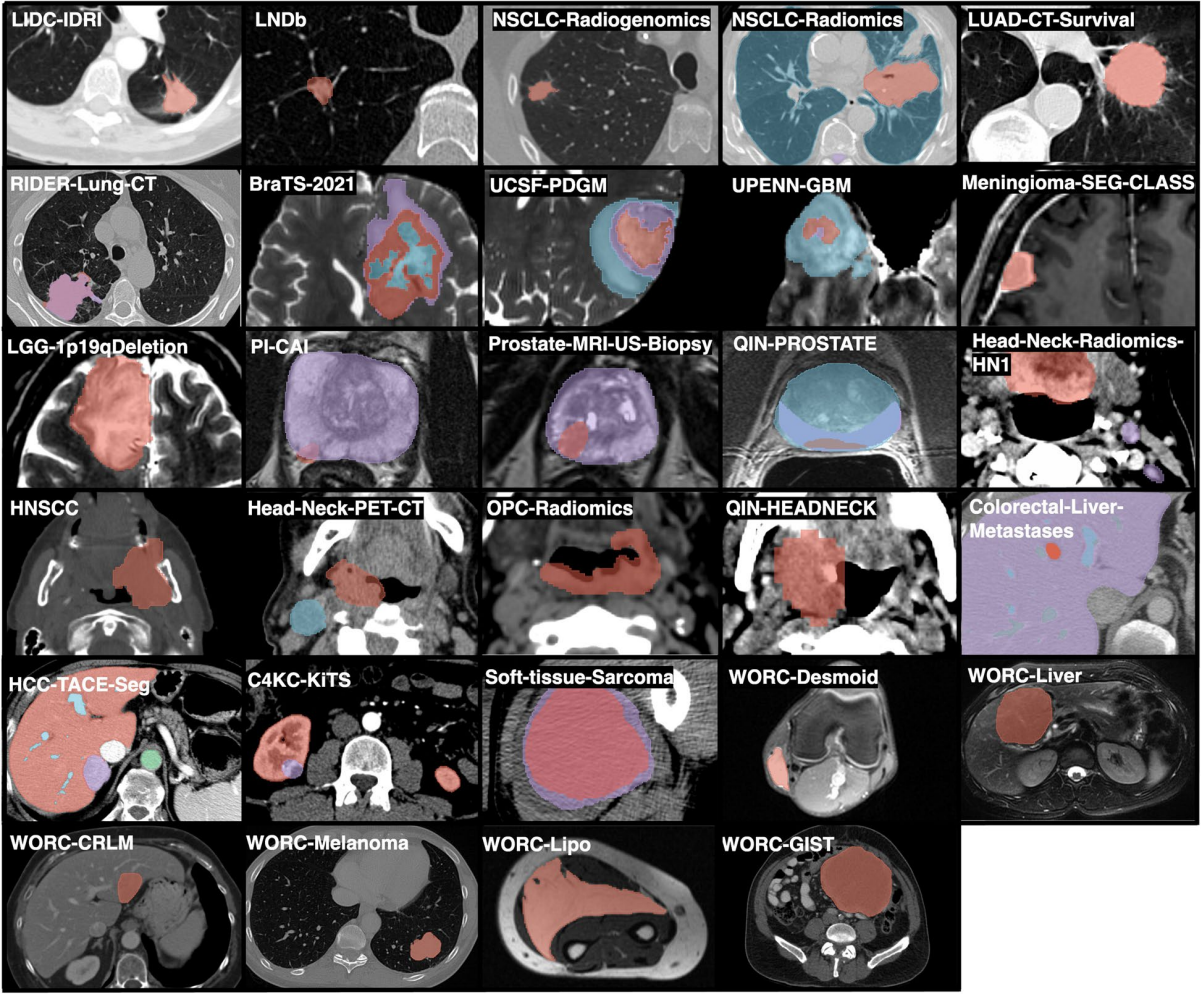
Examples of regions of interest from each dataset, demonstrating the diversity in imaging modalities and disease focuses

Out of 143 open-access datasets reviewed, we identified 29 datasets suitable for radiomics analysis, covering a wide range of cancer types and imaging modalities. The datasets encompassed 10,354 patients, 15,221 studies, and 49,515 scans. The most common organ of interest was the lung (7 datasets), followed by the head and neck (6 datasets), the brain (5 datasets), the prostate, the liver, and the soft tissue (each region was the focus of 3 datasets). A single dataset represented the gastrointestinal tract and kidney tumors. Table 1 presents the core statistics of the datasets, including the clinical tasks and imaging modalities used. The tasks ranged from binary classification (15 datasets) and multi-class classification (1 dataset) to survival analysis (11 datasets) and repeatability assessments (3 datasets). The most common imaging modality was computed tomography (CT), followed by magnetic resonance imaging (MRI) and positron emission tomography (PET/CT). Figure 2 showcases the diversity of imaging modalities and disease focuses through representative ROIs from each dataset.

**Table 1:** Core statistics of the datasets, including clinical tasks and imaging modalities used

Dataset name	Task	Task type	Imaging modality
LIDC-IDRI [20, 21]	Lung nodule classification	Binary classification	CT
LNDb [22, 23]	Lung nodule classification (conformant to LIDC-IDRI)	Multi-class classification	CT
NSCLC-Radiogenomics [24, 25]	Outcome prediction for non-small cell lung cancer	Survival analysis	CT, PET/CT
NSCLC-Radiomics [2]	Outcome prediction for non-small cell lung cancer	Survival analysis	CT
LUAD-CT-Survival [26, 27]	Classification of lung cancer patients into long/short survival	Binary classification	CT
RIDER-Lung-CT [2]	Repeatability of radiomics features for non-small cell lung cancer	Repeatability	CT
BraTS-2021 [28]	Classification of MGMT promoter methylation status in brain tumor	Classification	MRI
UCSF-PDGM [29]	Classification of MGMT promoter methylation and IDH mutation status, and outcome prediction in brain tumor	Classification, survival analysis	MRI
UPENN-GBM [30]	Outcome prediction for glioblastoma	Survival analysis	MRI
Meningioma-SEG-CLASS [31]	Meningioma grading (grade I vs. II)	Classification	MRI
LGG-1p19qDeletion [32]	Classification of 1p/19q co-deletion status of low-grade glioma	Classification	MRI
PI-CAI [33]	Detection of clinically significant prostate cancer	Classification	MRI
Prostate-MRI-US-Biopsy [34]	Detection of clinically significant prostate cancer	Classification	MRI
QIN-PROSTATE [35, 36]	Repeatability of radiomics features in patients with prostate cancer	Repeatability	MRI
Head-Neck-Radiomics-HN1 [2]	Outcome prediction for head and neck squamous cell carcinoma	Survival analysis	CT
HNSCC [37, 38]	Outcome prediction for head and neck squamous cell carcinoma	Survival analysis	CT
Head-Neck-PET-CT [39]	Outcome prediction for head and neck cancers	Survival analysis	PET/CT
OPC-Radiomics [40]	Outcome prediction for oropharynx cancer	survival analysis	CT
QIN-HEADNECK [41]	Repeatability of radiomics features for head and neck cancers before and after therapy	Repeatability	PET/CT
Colorectal-Liver-Metastases [42]	Pre-operative outcome prediction for colorectal liver metastases	Survival analysis	CT
HCC-TACE-Seg [43]	Outcome prediction for hepatocellular carcinoma (HCC) treated with transarterial chemoembolization (TACE)	Survival analysis	CT
C4KC-KiTS [44]	Kidney tumor segmentation and outcome prediction	Survival analysis	CT
Soft-tissue-Sarcoma [45]	Lung metastasis detection for sarcoma of the extremity	Binary classification	PET/CT, MRI
WORC-Desmoid [46, 47]	Classification of desmoid-type fibromatosis vs. extremity soft tissue sarcoma	Classification	MRI
WORC-Liver [46, 47]	Classification of malignant vs. benign primary solid liver tumor	Classification	MRI
WORC-CRLM [46, 47]	Classification of desmoplastic vs. replacement growth pattern in colorectal liver metastases	Classification	CT
WORC-Melanoma [46, 47]	Classification of BRAF-mutated vs. BRAF-wild in lung metastases of melanoma	Classification	CT
WORC-Lipo [46, 47]	Classification of well-differentiated liposarcoma vs. lipoma	Classification	MRI
WORC-GIST [46, 47]	Classification of gastrointestinal stromal tumor (GIST) vs. tumor resembling GIST	Classification	CT

### Data formats and annotation methods

Table 2 provides an overview of the image and segmentation formats used in the datasets and the segmented ROIs and annotation types. The original image formats included DICOM, NifTI, and MetaImage. The segmentation formats comprised DICOM Segmentation object (DICOM-SEG), DICOM Radiation Therapy Structure set (DICOM-RT), NifTI, MetaImage, and Stereolithography (STL) format. The primary ROIs varied across datasets and included typically tumor region. However, a few datasets had additional segmentations available, for organs of interest (lung, prostate, liver, kidney). Included datasets utilized manual, semiautomatic, and automatic segmentation techniques. Manual segmentations were performed by expert radiologists and radiation oncologists, while automatic segmentation methods employed state-of-the-art algorithms based on convolutional neural networks, such as U-Net [48] and its variants. Three datasets included segmentations from multiple readers.

**Table 2:** Overview of image and segmentation formats as well as segmentation region of interest (ROI), imaging phase or sequence and annotation type

Dataset name	Original image format	Original segmentation format	Primary ROI(s)	Phase/sequence	Annotation type
LIDC-IDRI	DICOM	DICOM-SEG	Lung nodule	Non-contrast	Manual (4 readers)
LNDb	MetaImage	MetaImage	Lung nodule	Non-contrast	Manual (1–3 readers)
NSCLC-Radiogenomics	DICOM	DICOM-SEG	Lung tumor	Non-contrast	Manual
NSCLC-Radiomics	DICOM	DICOM-SEG	Lung tumor	Non-contrast	Manual
LUAD-CT-Survival	DICOM	NIfTI	Lung tumor	Contrast-enhanced	Semiautomatic
RIDER-Lung-CT	DICOM	DICOM-SEG, DICOM-RT	Lung tumor	Non-contrast	Manual (radiation oncologist), automatic
BraTS-2021	NIfTI	NIfTI	Brain tumor	T1, T1CE, T2, FLAIR	Manual (4 readers)
UCSF-PDGM	NifTI	NifTI	Brain tumor	T2w, T2/FLAIR, SWI, DWI, T1w, T1CE, ASL, HARDI	Semiautomatic
UPENN-GBM	NIfTI	NIfTI	Brain tumor	T1w, T1CE, T2w, FLAIR, DTI, DSC	Manual, automatic
Meningioma-SEG-CLASS	DICOM	DICOM-RT	Brain tumor	T1w, T1CE, T2-FLAIR	Manual (radiation oncologist)
LGG-1p19qDeletion	DICOM	DICOM-SEG	Brain tumor	T1w, T2w	Manual
PI-CAI	MHA	NIfTI	Prostate lesion	T2w, ADC, high b-val (HBV)	Manual, automatic
Prostate-MRI-US-Biopsy	DICOM	STL	Prostate lesion	T2w	Manual
QIN-PROSTATE	DICOM	DICOM-SEG	Prostate lesion	T2w, ADC, DCE subtraction	Manual
Head-Neck-Radiomics-HN1	DICOM	DICOM-SEG	Head and neck tumor	Venous phase	Manual
HNSCC	DICOM	DICOM-SEG	Head and neck tumor	Non-contrast	Manual (radiation oncologist)
Head-Neck-PET-CT	DICOM	DICOM-RT	Head and neck tumor	Non-contrast (CT), static (PET)	Manual
OPC-Radiomics	DICOM	DICOM-SEG	Head and neck tumor	Non-contrast	Manual
QIN-HEADNECK	DICOM	DICOM-SEG	Head and neck tumor	Non-contrast (CT), static (PET)	Manual
Colorectal-Liver-Metastases	DICOM	DICOM-SEG	Liver tumor	Portal venous phase	Semiautomatic
HCC-TACE-Seg	DICOM	DICOM-SEG	Liver tumor	Portal venous phase	Semiautomatic
C4KC-KiTS	DICOM	DICOM-SEG	Kidney tumor	Corticomedullary phase	Manual
Soft-tissue-Sarcoma	DICOM	DICOM-RT	Soft tissue tumor	T1w, STIR, T2FS (MRI), non-contrast (CT), static (PET)	Manual
WORC Desmoid	NIfTI	NIfTI	Soft tissue lesion	T1w	Manual
WORC Liver	NIfTI	NIfTI	Liver lesion	T2w	Manual
WORC CRLM	NIfTI	NIfTI	Liver lesion	Portal venous phase	Manual
WORC Melanoma	NIfTI	NIfTI	Lung tumor	Contrast-enhanced	Manual
WORC Lipo	NIfTI	NIfTI	Soft tissue lesion	T1w	Manual
WORC GIST	NIfTI	NIfTI	GI tract lesion	Venous phase	Manual

### Detailed dataset description

The data sources, study times licensing, and cohort sizes are presented in Table 3. The study times ranged from the early 1990s to 2021, with 15 studies finishing after 2013. Most datasets were licensed under Creative Commons licenses (3.0 and 4.0), permissive of non-commercial and commercial usage and redistribution, and some datasets had custom or restricted licenses. The number of patients in the datasets varied from 15 to 1476, with the number of studies ranging from 30 to 11,523 and the number of scans ranging from 62 to 7,380. The largest dataset, in terms of patients, was the PI-CAI dataset (*n* = 1476) for detecting clinically significant prostate cancer using MRI. The LIDC-IDRI dataset, which focuses on lung nodule classification using CT, contained 1010 patients and 1308 studies. UCSF-PDGM dataset with brain tumor MRI cases included the most scans (*n* = 11,523) for 495 patients, which can be attributed to multiple sequences, including T2w, FLAIR, SWI, DWI, T1w, T1CE, ASL, and HARDI.

**Table 3:** Overview of dataset times, sources, licensing and cohort sizes *(CC—Creative Commons)*

Dataset name	Study time	Source	License	*n* patients	*n* studies	*n* series
LIDC-IDRI	2003–2011	TCIA	CC 3.0	1010	1308	1308
LNDb	2016–2018	grand-challenge	CC 4.0	236	236	236
NSCLC-Radiogenomics	1990–1995	TCIA	CC 3.0	211	303	1355
NSCLC-Radiomics	2004–2011	TCIA	CC 3.0	422	422	1265
LUAD-CT-Survival	2000–2011	TCIA	CC 3.0	40	40	40
RIDER-Lung-CT	2007	TCIA	CC 3.0	31	62	62
BraTS-2021	2012–2021	synapse/kaggle	custom^a^	1251	1251	5004
UCSF-PDGM	2015–2021	TCIA	CC 4.0	495	501	11,523
UPENN-GBM	2006–2018	TCIA	CC 4.0	630	3301	3601
Meningioma-SEG-CLASS	2010–2019	TCIA	TCIA restricted	96	180	674
LGG-1p19qDeletion	2002–2011	TCIA	TCIA restricted	159	160	478
PI-CAI	2012–2021	grand-challenge	CC 4.0	1476	1476	7380
Prostate-MRI-US-Biopsy	2010–2011	TCIA	CC 4.0	842	842	842
QIN-PROSTATE	2013–2015	TCIA	CC 4.0	15	30	270
Head-Neck-Radiomics-HN1	2000–2006	TCIA	TCIA no commercial	137	137	486
HNSCC	2005–2012	TCIA	TCIA restricted	627	1177	4039
Head-Neck-PET-CT	2006–2014	TCIA	TCIA restricted	298	504	2661
OPC-Radiomics	2005–2010	TCIA	TCIA restricted	606	606	1220
QIN-HEADNECK	2004–2013	TCIA	TCIA restricted	279	1032	3837
Colorectal-Liver-Metastases	2003–2007	TCIA	CC 4.0	197	197	394
HCC-TACE-Seg	2002–2012	TCIA	CC 4.0	105	214	677
C4KC-KiTS	2010–2018	TCIA	CC 3.0	210	210	621
Soft-tissue-Sarcoma	2004–2011	TCIA	CC 3.0	51	102	612
WORC Desmoid	1990–2018	XNAT	Custom^b^	203	203	203
WORC Liver	2002–2018	XNAT	custom^b^	186	186	186
WORC CRLM	2003–2015	XNAT	custom^b^	77	77	77
WORC Melanoma	2012–2018	XNAT	custom^b^	103	103	103
WORC Lipo	2009–2018	XNAT	custom^b^	115	115	115
WORC GIST	2004–2017	XNAT	custom^b^	246	246	246

^a^“Free to use and/or refer to the BraTS datasets in your own research” with citation

^b^License similar to CC 4.0 but no redistribution

### Clinical labels and predictors

Table 4 describes labels and clinical predictors provided for each dataset. The labels included health outcomes (overall survival, recurrence- and progression-free survival), pathologic tumor type and grade, TNM status, genetic markers, and imaging-based scores. Clinical predictors varied across datasets, including demographic information (age, sex, BMI), medical history (risk factors), laboratory parameters, clinical scores, and treatment details.

**Table 4:** A detailed description of dataset labels and clinical predictors

Dataset name	Type of labels	Clinical predictors
LIDC-IDRI	Malignancy diagnosis (patient-level and nodule-level)	-
LNDb	Fleischner score	-
NSCLC-Radiogenomics	Survival data, TNM status (for selected cases)	Age, weight, gender, ethnicity, smoking status, tumor histology, treatment (yes/no)
NSCLC-Radiomics	Overall survival, TNM status	Age, gender, tumor histology
LUAD-CT-Survival	Survival class (top quartile—long survival/lowest quartile—short survival)	-
RIDER-Lung-CT	Not available—repeatability study	-
BraTS-2021	MGMT promoter methylation status	-
UCSF-PDGM	MGMT promoter methylation status, IDH mutation status, overall survival	Age
UPENN-GBM	Overall survival, MGMT promoter methylation status, IDH mutation status, progression score	Age, gender
Meningioma-SEG-CLASS	Pathologic grade	Age, sex, tumor subtype, atypical features
LGG-1p19qDeletion	1p/19q deletion status	Tumor histology
PI-CAI	Gleason score (csPCa vs. non-csPCa)	Age, PSA, PSAD, prostate volume
Prostate-MRI-US-Biopsy	Gleason score (csPCa vs. non-csPCa)	-
QIN-PROSTATE	Not available—repeatability study	-
Head-Neck-Radiomics-HN1	Overall survival, recurrence-free survival, time to local/locoregional/distant recurrence, TNM status, AJCC stage	Age, sex, HPV p16 status, pretreatment Hb concentration, detailed treatment
HNSCC	Overall survival, recurrence-free survival, time to local/regional/locoregional/distant recurrence, TNM status, AJCC stage	Age, gender, smoking status, HPV status, detailed treatment
Head-Neck-PET-CT	Overall survival, time to locoregional recurrence, time to distant metastasis, TNM status	Age, sex, HPV status, therapy type
OPC-Radiomics	Overall survival, time to local/regional/distant failure, TNM status	Age, sex, ECOG performance status, smoking status, alcohol status, tumor histology
QIN-HEADNECK	Overall survival with cause of death, recurrence-free survival with recurrence location, TNM status	Age, sex, weight, height, race, medical history, alcohol status, smoking status, treatment info
Colorectal-Liver-Metastases	Overall survival, time to disease progression, time to liver disease progression	Age, sex, NASH score, total response (%), necrosis (%), fibrosis (%), mucin (%)
HCC-TACE-Seg	Overall survival and time-to-progression, TNM status	Age, sex, smoking status, alcohol status, HBV/HCV status, medical history, pathology differentiation status, metastasis presence, vascular invasion, AFP level, CLIP score, detailed therapy, EASL, RECIST
C4KC-KiTS	Overall survival, TNM status, ISUP grade	Age, gender, BMI, comorbidities, smoking status, alcohol status, tumor histology subtype, detailed treatment, eGFR preop and postop
Soft-tissue-Sarcoma	Overall survival, disease-free survival (with recurrence/metastasis location)	Age, sex, tumor histology, treatment info
WORC Desmoid	Binary (fibromatosis vs. sarcoma)	Age, sex
WORC Liver	Binary (malignant vs. benign lesion)	Age, sex
WORC CRLM	Binary (histological growth type)	Age, sex
WORC Melanoma	Binary (BRAF mutated vs. BRAF wild)	Age, sex
WORC Lipo	Binary (liposarcoma vs. lipoma)	Age, sex
WORC GIST	Binary (GIST vs. non-GIST)	Age, sex

### Radiomics features

**Fig. 3:**
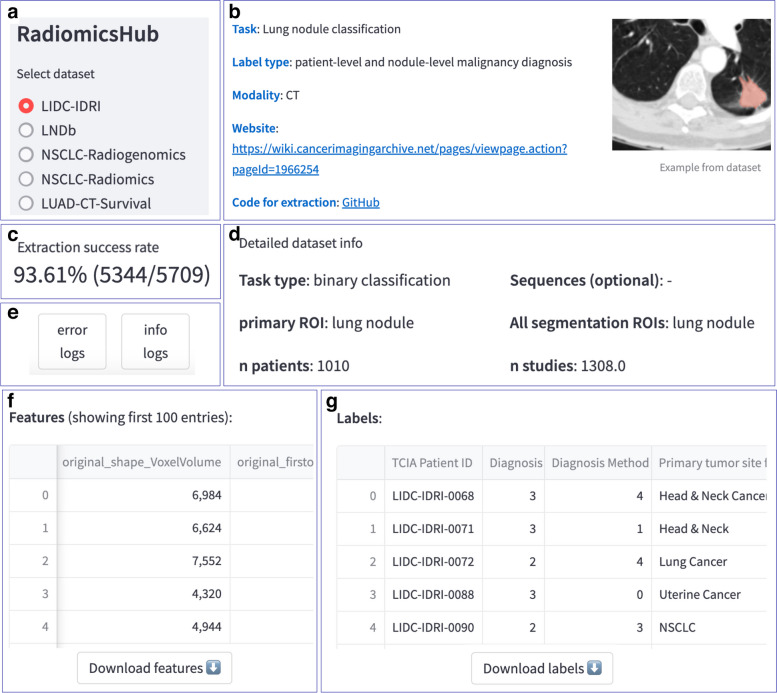
View of metadata and extraction artifacts for a selected dataset (LIDC-IDRI). **a** Dropdown menu for dataset selection, **b** most important dataset information, **c** extraction success rate, **d** detailed dataset information, **e** logs for download, **f** radiomics features, **g** labels. An interactive version of the wiki is available at https://radiomics.uk

**Fig. 4:**
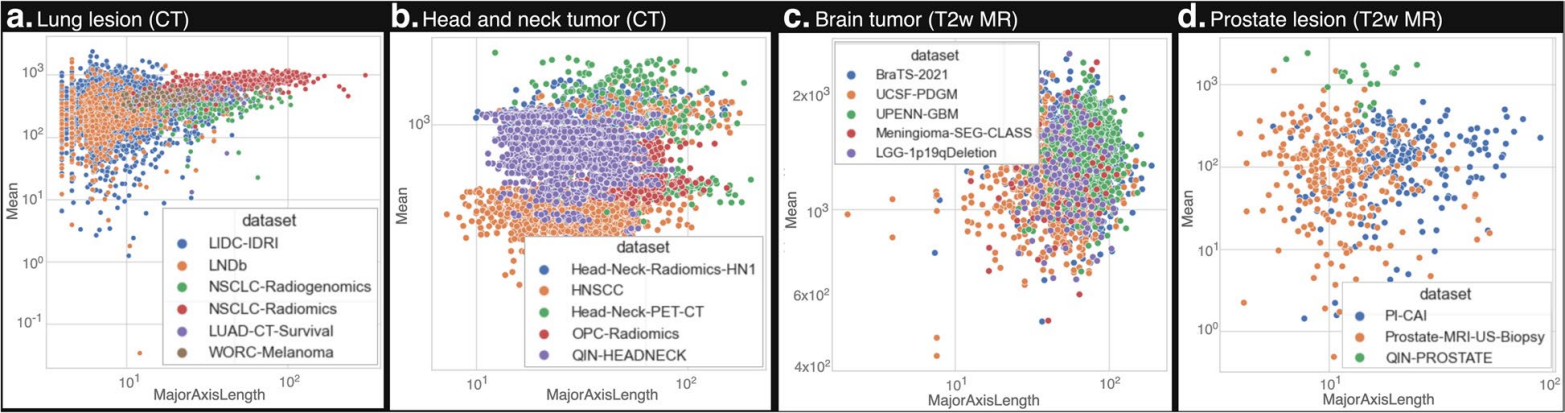
Scatterplot illustrating the relationship between core radiomics features. Mean intensity and major axis length for shared regions of interest and modality are plotted across multiple datasets. The substantial overlap observed in the feature distributions suggests the feasibility of merging these datasets for a comprehensive evaluation

All datasets were successfully preprocessed and radiomics features were extracted with specified settings. The results of the preprocessing and extraction for each dataset are available online at https://radiomics.uk, with an overview of this website provided in Fig. 3. The website presents each dataset with its detailed metadata, examples, links to sources, code used for extraction, and logs. Its core is the tables with radiomics features and labels available for download. It also includes a form that allows the user to request a new dataset. We also investigated the association between core radiomic features: mean intensity and major axis length across overlapping regions of interest and imaging modalities in our collection. Figure 4 shows a significant overlap in the distribution of these features, which emphasizes the potential for integrating multiple datasets for a more extensive evaluation.

## Discussion

In this study, we introduced RadiomicsHub, a repository and a wiki designed to streamline the utilization of open-access cancer imaging datasets for radiomics research. The primary goal of RadiomicsHub is to enable the efficient evaluation of novel radiomics models on benchmark datasets, addressing the time-consuming task of locating appropriate datasets with segmentations and outcomes/labels and preprocessing them from their raw form. Our detailed examination of publicly available datasets revealed a collection well-suited for radiomics research. The key findings present a great diversity in imaging modalities, data formats, segmentation techniques, clinical labels, and predictors across the datasets, with comprehensive details and associated radiomics features made readily accessible online.

By converting all datasets into a common format (NifTI) and making the conversion process reproducible and traceable, RadiomicsHub ensures consistency and reliability in the data used for model evaluation. Furthermore, the extraction of radiomics features from each dataset, using various parameter settings, and the availability of metadata and descriptions online contribute to a comprehensive and accessible platform for researchers, which may serve as a bridge between nuanced radiomics research and practical, clinical oncology care. Importantly, standardized, and processed radiomics data are invaluable for developing robust machine learning models trained on high-quality, validated public datasets. As a rich, curated repository of radiomics features, RadiomicsHub emerges as a potential catalyst in translating radiomics research findings into tangible clinical applications. We are committed to maintaining and expanding the project in collaboration with the research community.

RadiomicsHub builds upon existing open science projects and repositories, such as the TCIA, Grand Challenge, and Zenodo, which have laid the foundation for sharing imaging datasets. A few other notable projects committed to promoting open science and collaboration exist in the domain of medical imaging. EUCanImage [49] is a consortium that is building a highly secure, federated, and large-scale cancer imaging platform across Europe, aimed at enhancing the use of AI in oncology. Although there are parallels in our goal to identify and utilize cancer imaging data, EUCanImage is a large initiative focusing on data exchange and storage. In contrast, our study focuses on providing the methods to preprocess and extract radiomics features that can be reproduced locally. The National Cancer Institute (NCI) Imaging Data Commons (IDC) [50] is a cloud-based platform that provides access to diverse cancer-related medical imaging datasets from various sources, including TCIA and other NCI-supported projects. It aims to facilitate the development and validation of AI models, computational models, and quantitative imaging methods by making it easier for researchers to find, access, and analyze large-scale imaging datasets. Open Access Series of Imaging Studies (OASIS) [51] is another project that offers a publicly accessible collection of neuroimaging data, including cross-sectional and longitudinal MRI data. Other large-scale initiatives contributing valuable imaging data to their respective research fields include Alzheimer’s Disease Neuroimaging Initiative (ADNI) [52], UK Biobank [53], and the German National Cohort (NAKO) [54] studies. ADNI focuses on collecting and sharing Alzheimer’s disease-related data, including MRI and PET images. The UK Biobank offers an extensive collection of genetic, lifestyle, and health data from half a million UK participants, including brain, cardiac, and abdominal MRI datasets. The NAKO study investigates the causes of chronic diseases by collecting a wealth of data, including imaging data, from a large German population.

Our study complements these initiatives by focusing on providing standardized and processed radiomics data, making it a specialized resource for the radiomics research community. As a living repository, it has the potential to grow and adapt to the evolving needs of the community by incorporating new datasets, feature sets, and tools reflecting the latest developments and innovations in the field. With a commitment to open science and a focus on collaborative research, we hope its results will stimulate further research and innovation within the research community, further expanding its scope and capabilities. We hope that through this dynamic nature, RadiomicsHub will remain relevant and valuable to researchers, fostering collaboration and accelerating the progress of radiomics research. We believe that pooling different datasets will spark interest in novel research questions, such as the impact of study-specific parameters (acquisition parameters, study time, annotation method, and quality) on the distribution of radiomics features and clinical variables.

While we have focused on the core features of RadiomicsHub, there are potential areas for expansion and improvement. For instance, allowing single images and segmentations to be downloaded through an API or providing TotalSegmentator [4] organ masks for CT datasets could enhance the platform’s utility. Additionally, offering baseline models for each dataset could assist researchers in comparing the performance of their models against established benchmarks. This could be achieved by using the recently published *AutoRadiomics* [18] framework. Additionally, feature harmonization methods, such as ComBat [55], could be used to compensate for multicenter effects affecting extracted radiomics features. ComBat can align feature distributions across different sites without performing any additional image processing. Adding this step to subsequent analyses would ensure models trained on our data work reliably in various settings, which is necessary for successful clinical translation.

There are potential risks and challenges associated with RadiomicsHub. One such concern is the possibility of introducing errors or generating non-meaningful processed data and features during the conversion and preprocessing steps. To address this concern, we have implemented robust quality control measures, including standard, reproducible processing instructions, and error logging. Volumes have been tested for various assertions, including correct dimensionality, shape, label presence, and valid ROI placement. However, despite our efforts, there remains a residual risk regarding the integrity and accuracy of the data.

## Conclusions

In this study, we developed a comprehensive repository with radiomics features from public cancer imaging datasets that can be readily used for robust evaluation of radiomics models. We addressed the challenges associated with dataset preprocessing and radiomics feature extraction, ensuring reproducibility and offering our scripts for reuse. We believe that fostering a collaborative research environment and promoting standardized datasets can accelerate the discovery of new biomarkers and improve clinical decision-making in oncology and beyond.

### Supplementary Information

**Additional file 1: S1. **Preprocessing details.** S2.** Feature extraction settings.

## Data Availability

The code and dataset(s) supporting the conclusions of this article is(are) available in the GitHub repository, https://github.com/pwoznicki/RadiomicsHub. The study artifacts, including radiomics features and labels, are publicly available at https://radiomics.uk. The original datasets analyzed in the study are publicly available from their referenced sources with licenses described in the current study. The GitHub repository includes software used for our analysis: Project name: RadiomicsHub. Project home page: https://github.com/pwoznicki/RadiomicsHub. Archived version: https://github.com/pwoznicki/RadiomicsHub/commit/802312fce88b0d13ad837023418d3ff578a17e8b. Operating system(s): Platform independent. Programming language: Python. Other requirements: listed in the repository. License: MIT.

## Abbreviations

ADNI: Alzheimer’s Disease Neuroimaging Initiative
AI: Artificial intelligence
CLEAR: CheckList for EvaluAtion of Radiomics Research
DICOM: Digital Imaging and Communications in Medicine
DICOM-RT: DICOM Radiation Therapy
DICOM-SEG: DICOM Segmentation object
IDC: Imaging Data Commons
NAKO: German National Cohort
NCI: National Cancer Institute
NIfTI: Neuroimaging Informatics Technology Initiative (format)
OASIS: Open Access Series of Imaging Studies
ROI: Region of interest
STL: Stereolithography (format)
TCIA: The Cancer Imaging Archive
