# Supplementary material for: Addressing challenges in radiomics research: systematic review and repository of open-access cancer imaging datasets
Source: Insights Imaging. 2023 Dec 12;14:216. doi: 10.1186/s13244-023-01556-w (PMC10716101; doi:10.1186/s13244-023-01556-w)
Supplement: Supplementary file 1 — Additional file 1: S1. Preprocessing details. S2. Feature extraction settings. [file 13244_2023_1556_MOESM1_ESM.docx]

**Addressing Challenges in Radiomics Research: Systematic Review and Repository of Open-Access Cancer Imaging Datasets**

**ELECTRONIC SUPPLEMENTARY MATERIAL**

**S1. Preprocessing details.**

Normalization was performed for MRI data because this modality inherently exhibits variable intensity values both within a single dataset and across different datasets. In contrast, CT and PET data typically adhere to standardized intensity scales. Images and segmentations were resampled to an isotropic resolution of *2mm x 2mm x 2mm*. For interpolation, the standard B-spline interpolator was used due to its ability to provide smooth transformations and accurately model non-linear deformations, which is important for maintaining tissue continuity after resampling.

**S2. Feature extraction settings.**

Filters: *original, Laplacian of Gaussian with sigma equal to 3.0 and 5.0, Wavelet*

Feature classes: *first-order, shape, GLCM, GLRLM, GLSZM, GLDM, NGTDM*

Bin width: *25*

Voxel array shift: *1000*

Kernel radius: *2*
